# Dietary contributions in the genetic variation of liver fibrosis: a genome-wide association study of fibrosis-4 index in the liver fibrosis development

**DOI:** 10.1186/s13578-024-01321-6

**Published:** 2024-11-22

**Authors:** Poppy Diah Palupi, Chun-Yu Wei, Wan-Hsuan Chou, Min-Rou Lin, Yu-Jui Yvonne Wan, Wei-Chiao Chang

**Affiliations:** 1 Department of Clinical Pharmacy, School of Pharmacy, College of Pharmacy, Taipei Medical University, Taipei, 11031 Taiwan; 2 Core Laboratory of Neoantigen Analysis for Personalized Cancer Vaccine, Office of R&D, Taipei Medical University, Taipei, 11031 Taiwan; 3 Department of Medical Pathology and Laboratory Medicine, University of California, Davis, Sacramento, CA USA; 4 Master Program in Clinical Genomics and Proteomics, Taipei Medical University, Taipei, 11031 Taiwan; 5 Integrative Research Center for Critical Care, Department of Pharmacy, Taipei Medical University-Wan-Fang Hospital, Taipei, 11696 Taiwan; 6 Department of Pharmacy, Wan Fang Hospital, Taipei Medical University, Taipei, 11696 Taiwan

**Keywords:** Fibrosis-4 index, GWAS, *HBS1L*, *HLA-DQB1*, Dietary intake, Taiwanese population

## Abstract

**Background:**

The fibrosis-4 (FIB-4) index is a non-invasive method to assess the severity of liver fibrosis. The development of liver fibrosis is influenced by genetic predisposition and dietary factors. However, the modulating effect of dietary factors on the genetic susceptibility of liver fibrosis remains unclear. The study aims to investigate the role of dietary factors in modulating the genetic susceptibility of liver fibrosis.

**Methods:**

Here, we conducted a genome-wide association study (GWAS) of FIB-4 index-directed liver fibrosis risk, adjusted with diet, lifestyle factors, and hepatitis serological markers. The high (*N* = 1,476) and low (*N* = 36,735) liver fibrosis risk groups were defined with a FIB-4 > 2.67 and < 1.3, respectively.

**Results:**

The age-related FIB-4 variation showed subjects with a FIB-4 > 2.67 (3.8%), indicating high fibrosis risk, occurred predominantly among individuals above 60 years old. The multivariable analysis showed that tea intake is significantly associated with a reduced risk of liver fibrosis. The GWAS adjusted for sex, age, age^2^, dietary factors (tea and coffee consumption, vegetarian preference), lifestyle (alcohol consumption, physical activity), hepatitis serological markers (anti-HCV, HBsAg, HBeAg), and the top ten principal components indicated 25 genome-wide significant signals (*p* < 5 × 10^− 8^). Two variants (rs56293029 and rs9389269) were previously associated with the FIB-4 index in alcohol-related cirrhosis, while the 23 SNPs remaining were novel. The rs9399136 (*HBS1L)* is a protective variant, and rs9274407 (*HLA-DQB1*) is a risk variant, both contributing to liver fibrosis development. Our results showed that genetic factors play a major role in liver fibrosis, while dietary factors have minor effects on disease progression. Pathway analysis suggested the potential of immune response and hematopoietic systems function in the pathogenesis of liver disease.

**Conclusions:**

The studies not only revealed the protective role of rs9399136 (*HBS1L)* and the risk effect of rs9274407 (*HLA-DQB1)* toward liver fibrosis in a Taiwanese population, but also demonstrated that individual consumption patterns, such as tea uptake, have a minor impact on liver fibrosis prevention. The pathway analysis from GWAS variants further indicated the importance of immune responses in the pathogenesis of liver fibrosis.

**Supplementary Information:**

The online version contains supplementary material available at 10.1186/s13578-024-01321-6.

## Introduction

Liver fibrosis is a chronic liver disorder that develops due to an abnormal wound-healing response, characterized by excessive amounts of scar tissue due to inflammation and injury [1]. Progressive liver fibrosis occurs gradually and might lead to cirrhosis, putting individuals at risk for liver cancer and increasing the likelihood of liver failure [2, 3]. Liver biopsy is a standard diagnosis of fibrosis prompted by elevated hepatic enzymes [4]. However, the invasive procedure has several complications, including patient reluctance, hypotension, infection, and potential bleeding [5]. Timely detection of liver fibrosis can avoid decompensation, liver cancer, and fatality [6]. Therefore, there is a need for accurate, reproducible, and simple methods to assess liver fibrosis [7]. Recently, non-invasive methods such as the Fibrosis-4 Index (FIB-4) have been widely used to predict the probability of liver dysfunction [8]. The techniques only require routine measurement of laboratory data, such as platelet count and aminotransferase serum levels. The European Association for the Study of the Liver (EASL) practice guideline strongly recommends using the FIB-4 Index to identify the probability of liver fibrosis in individuals with metabolic risk factors and alcohol users [9]. A study on alcohol-related cirrhosis in the European population identified variants associated with the FIB-4 index at *MRC1*,* HBS1L*, and *ARHGEF3* gene region [10]. Moreover, FIB-4 is a straightforward method of identifying patients at high risk of severe liver fibrosis in the community setting [11].

Diet significantly influences liver health and impacts the risk of fibrosis. A nutritious diet rich in low-fat dairy products, white meat, nuts, vegetables, fruits, and vegetable oils, and moderate coffee and tea consumption could potentially mitigate liver inflammation and fibrosis progression [12]. Given the significant role of diet in liver health, it is essential to understand how genetic factors interact with dietary factors to influence fibrosis risk. Dietary factors such as tea and coffee consumption can affect liver health through their anti-inflammatory and antioxidant properties [13]. Nutritional factors like tea and coffee, rich in polyphenols, may interact with genetic variants to reduce oxidative stress and modulate the risk of liver fibrosis [14].

Although the FIB-4 index is widely used, the distribution of the FIB-4 index in the Taiwanese is unknown. Moreover, the genetic basis underlying liver fibrosis remains unclear. Thus, we calculated the FIB-4 index for 38,211 individuals and analyzed susceptible genetic risk loci of liver fibrosis, considering the potential modifying effects of diets, lifestyle factors and hepatitis markers on genetic risk factors. This approach aims to provide insight into the genetic and dietary influences on liver fibrosis risk in the Taiwanese population.

## Materials and methods

### Study population and phenotype selection

The current study uses data obtained from Taiwan Biobank (TWB). The studied cohort includes 68,948 individuals aged between 30 and 70. The study received ethical approval from the Institutional Review Board (IRB no. N201906005) of Taipei Medical University prior to its commencement. The FIB-4 distribution was stratified based on age group, which is > 50, 50–59, and > 60 years. The age classification was determined by assessing the risk of MASH (metabolic dysfunction-associated steatohepatosis) in individuals > 50 years, with confirmed fibrosis identified specifically among subjects 60 years or older [15, 16]. We used PLINK 2.0 to perform individual quality control (QC). The exclusion criteria include sample call rate below 98%, sex discrepancy, heterozygosity rate that deviated from the mean ± 3SD, and second-degree relatives based on the identity by descent (IBD) above 0.1875. The standard QC process resulted in 59,448 individuals for final analysis. The FIB-4 index was estimated based on the participant’s clinical data using the formula = Age [years] × AST [U/L] / platelet [10^9^/L] × ALT^1/2^ [U/L] [8]. Participants were further classified as [1] low-risk of liver fibrosis, FIB-4 < 1.3; [2] intermediate-risk, 2.67 ≥ FIB-4 ≥ 1.3; [3] high-risk, FIB-4 > 2.67 [17]. Individuals with a high risk of fibrosis (*n* = 1,476 subjects) were defined as cases, and individuals with a low risk of fibrosis (*n* = 36,735) were defined as controls.

### Genotyping, imputation, and QC filtering

Genotyping was conducted using an Affymetrix^®^ TWB chip (Axion Genome-Wide Array Plate). Imputation was performed with IMPUTE2 v2.3.1 software based on a Taiwan Biobank reference panel and the 1000 Genome Project of the East Asian (EAS) population. A total of 16,222,535 SNPs were yielded after imputation. Post-imputation QC was performed using PLINK v2.0. The SNP exclusion criteria include: [1] variant call rate below 98% [2], minor allele frequency (MAF) of less than 5%, and [3] Hardy-Weinberg equilibrium *p-value* less than 1 × 10^− 6^. Insertions and deletions (INDELs) were also excluded. After QC, a total of 3,636,444 SNPs were left for the study.

### SNP heritability analysis

The SNP heritability was assessed utilizing LDAK SumHer [18], with estimation performed under the Human Default Model. The tagging file was obtained by calculating the expected heritability tagged by each predictor. The SNP heritability assesses the extent to which available genetic variations impact phenotypes, thereby enhancing the comprehension of the genetic framework governing complex traits. A summary statistic from each predictor was employed.

### Dietary and lifestyle information

The dietary factors (tea consumption status, coffee consumption status, and vegetarian status) and lifestyle factors (physical activity status and alcohol consumption status) included in this study were based on binary indicators obtained from the self-report questionnaire. Tea consumption status is defined positive as drinking tea at least once per day for a minimum of 6 months. Furthermore, coffee consumption status is positive if a participant consumes coffee at least three times per week for a minimum of 6 months. A positive vegetarian status was defined as being vegan for at least 6 months. Moreover, a positive physical activity status was defined as engaging in exercise at least three times per week, with each session lasting more than 30 min. Alcohol consumption status was considered positive if intake was at least 150 cc per week for 6 months. In addition, the serological status of the hepatitis profiles was assessed using key markers: anti-HCV (for hepatitis C exposure), HBsAg (for active hepatitis B infection), and HBeAg (for HBV replication status).

### Statistical analysis

A multivariate logistic regression analysis was conducted to investigate the factors associated with the risk of liver fibrosis. The independent variables included in the model were age, sex, tea and coffee consumption, vegetarian preference, alcohol intake, physical activity, and serological status of hepatitis (anti-HCV, HBsAg, and HBeAg). Age was considered as a continuous variable, while sex, tea, alcohol, coffee intake, vegetarian preference, physical activity, and hepatitis serological status were categorical variables. The dependent variable was the FIB-4 directed liver fibrosis risk. This multivariate logistic regression model provided adjusted odds ratio (OR) and 95% confidence intervals (CIs) to assess the independent effect of each covariate, with a significance level set at *p* < 0.05. The analysis was performed using a generalized linear model (GLM) in R. Following this association, a case-control GWA analysis of the FIB-4 index-directed liver fibrosis risk was performed to explore the genetic variants accounting for nutritional intake. Age, age^2^, sex, tea, coffee consumption, vegetarian preference, alcohol intake, physical activity, hepatitis serological markers, and the top ten principal components were included as covariates in the GWAS. The association analysis was performed by PLINK 2.0. The variants were considered genome-wide significant if they passed the *p-value* threshold of 5 × 10^− 8^. The suggestive significance was specified at the *p-value* of 1 × 10^− 5^. Manhattan plot was generated using the CMPlot package and quantile-quantile plots using the qqman package in R studio. We applied Conditional and Joint (COJO) analysis in GCTA v1.94.1 using a stepwise model selection method to select independent signals. The analysis aims to identify the causal variants within the genomic region and clarify the specific variants that contribute independently to the observed association. The variants were reported if located in the genomic areas within ± 10 Mb with minor allele frequency > 0.01, collinearity cut-off was 0.9, and reached genome-wide significance (*p* < 5 × 10^− 8^). Variants with *r-values* close to zero indicate small or no correlation. The regional plot of association signals near the lead SNPs was generated using LocusZoom (http://locuszoom.org/).

### Functional annotation and pathway enrichment analysis

ANNOVAR [19] was utilized for functional annotation. The ClusterProfiler package in R was used to perform functional enrichment analysis to determine the biological function of the genes in suggestive variants. Pathways with a *false discovery rate (FDR)* < 0.05 were considered significant. A comprehensive enrichment analysis was conducted on 84 variants using data from gene ontology (GO): biological processes (GO: BP), Reactome, and KEGG. The bubble plot was used to visualize the pathway enrichment in each database.

## Results

### The distribution of the FIB-4 index in a Taiwanese population

In the general population of Taiwan, the mean ± SD of the FIB-4 index was 0.74 ± 0.21. In the context of age-related variation in FIB-4 values, it was noted that individuals aged > 60 years displayed a higher FIB-4 index of 1.67 ± 1.37, in comparison to the FIB-4 index of 1.12 ± 0.76 seen in 50–59 years. The lowest FIB-4 index of 0.81 ± 0.37 was observed in individuals below 50 (Table 1). Notably, the FIB-4 index ≥ 2.67 indicates a high risk of liver fibrosis. As shown in Table 1, individuals over 60 years old exhibited a high risk of liver fibrosis, constituting 33% of those with FIB-4 index exceeding 2.67. The demography of the cohort is shown in Supplementary Fig. 1.

**Table 1:** The distribution of the FIB-4 index in the general population of Taiwan

Age group (years)	*N*	FIB-4 index				
		Mean ± SD	Median	Min-max	FIB-4 < 1.30 *N* (%)	FIB-4 > 2.67 *N* (%)
< 50	22,930	0.81 ± 0.37	0.78	0.11–26.39	22,840 (99.6)	90 (0.4)
50–59	10,747	1.12 ± 0.76	1.05	0.32–43.54	10,379 (96.6)	368 (3.4)
≥ 60	4,533	1.67 ± 1.37	1.19	0.36–34.45	3,515 (77)	1,018 (33)
Overall	38,211	0.74 ± 0.21	0.92	0.11–43.54	36,735 (96.2)	1,476 (3.8)

### Diet consumption factors associated with the risk of liver fibrosis

The multivariable logistic regression analysis results indicate that tea consumption is significantly associated with a decreased risk of liver fibrosis (OR = 0.52, 95% CI = 0.36–0.74). Similarly, coffee intake shows a slightly reduced risk of liver fibrosis (OR = 0.80, 95% CI = 0.58–1.09), although the result is not statistically significant. Additionally, vegetarianism appears to be linked with an increased risk of liver fibrosis (OR = 1.44, 95% CI = 0.88–2.25), but this finding didn’t reach statistically significant. In addition to the diet variables, we incorporated lifestyle factors, including alcohol consumption, physical activity, and hepatitis viral profiles, into the analysis. The alcohol intake was significantly associated with an increase in the risk of liver fibrosis (OR = 2.19, 95% CI = 1.74–2.75), while routine physical activity is associated with a lower risk of liver fibrosis (OR = 0.77, 95% CI = 0.68–0.87). The hepatitis serological status, including anti-HCV, HBsAg, and HBeAg, also demonstrated as a significant increased risk for liver fibrosis with the following odds ratio: anti-HCV (OR = 5.59, 95% CI = 4.39–7.09), HBsAg (OR = 4.05, 95% CI = 3.43–4.78), and HBeAg (OR = 3.33, 95% CI = 1.79–5.92) (Table 2).

**Table 2:** Multivariable logistic regression analysis in risk of liver fibrosis

Variables	Estimated	OR (95%CI)	Standard error	*p*-value
Sex	-0.322	0.72 (0.64–0.82)	0.064	6.57 × 10 ^− 07^
Age	0.225	1.25 (1.24–1.26)	0.005	2.00 × 10 ^− 16^
Tea consumption	-0.654	0.52 (0.36–0.74)	0.183	3.56 × 10 ^− 04^
Coffee consumption	-0.219	0.80 (0.58–1.09)	0.160	1.70 × 10 ^− 01^
Vegetarianism	0.364	1.44 (0.88–2.25)	0.238	1.26 × 10 ^− 01^
Alcohol consumption	0.786	2.19 (1.74–2.75)	0.116	1.29 × 10 ^− 11^
Physical activity	-0.262	0.77 (0.68–0.87)	0.062	2.57 × 10^− 5^
Anti-HCV	1.721	5.59 (4.39–7.09)	0.122	2.00 × 10 ^− 16^
HBsAg	1.399	4.05 (3.43–4.78)	0.084	2.00 × 10 ^− 16^
HBeAg	1.203	3.33 (1.79–5.92)	0.303	7.45 × 10^− 5^

### GWAS of liver fibrosis risk

**Fig. 1:**
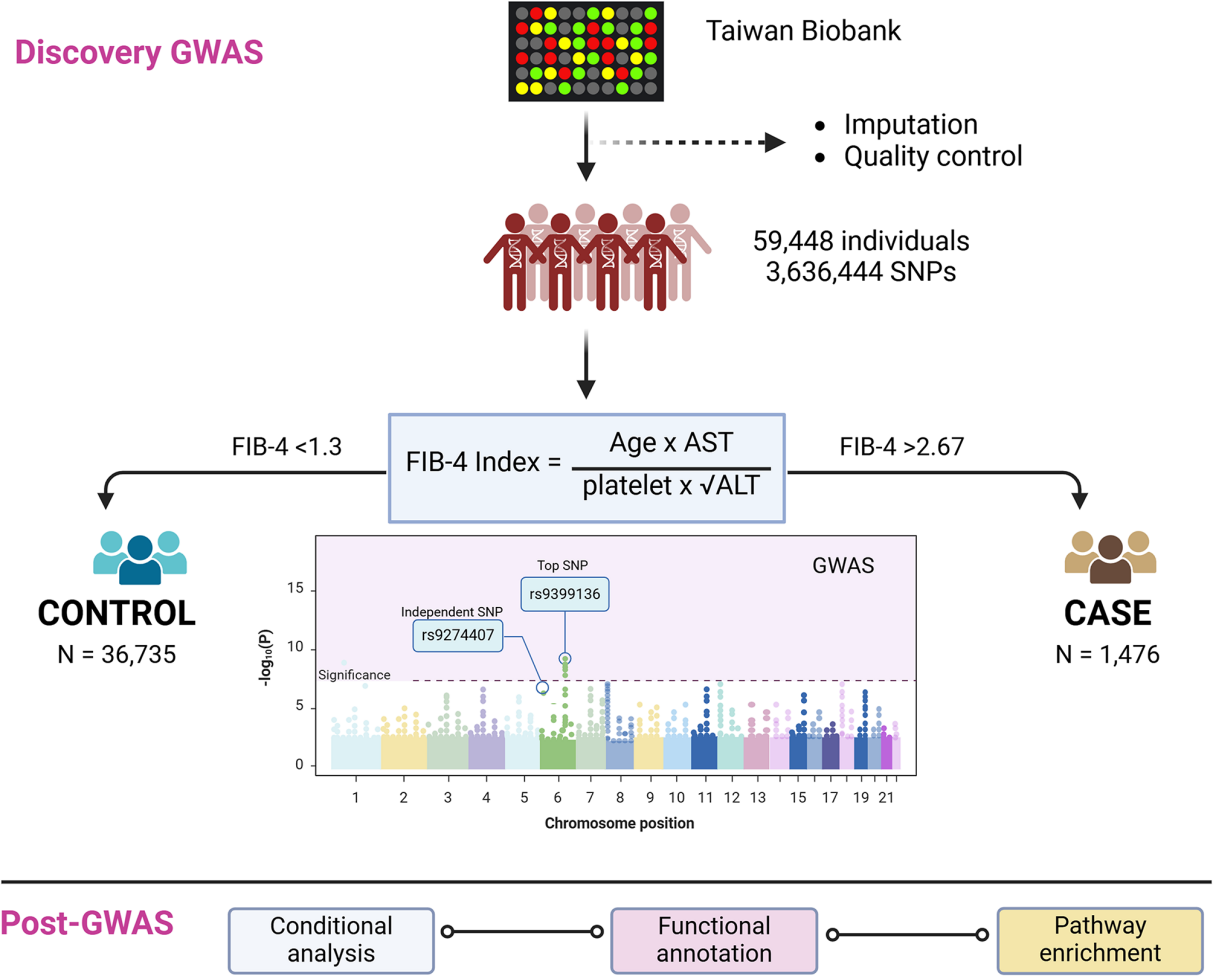
Workflow diagram and study design. FIB-4 Index, Fibrosis-4; AST, aspartate transaminase; ALT, alanine transaminase; GWAS, genome-wide association study (Created with Biorender https://app.biorender.com/*)*

We further conducted a genome-wide association analysis to identify variants associated with FIB-4 in the Taiwanese population. The cases were defined as those with high-risk (FIB-4 > 2.67, *n* = 1,476), and the controls were characterized as those with low-risk (FIB-4 < 1.3, *n* = 36,735) for liver fibrosis. This analysis was conducted across 3,636,444 variants. The clinical attributes of the study participants have been outlined in Supplementary Table 1. The study workflow and design are illustrated in Fig. 1. The quantile-quantile plot with the genomic factor (λgc) rate of 1.05 indicated the absence of population stratification, depicted in Supplementary Fig. 2.

**Fig. 2:**
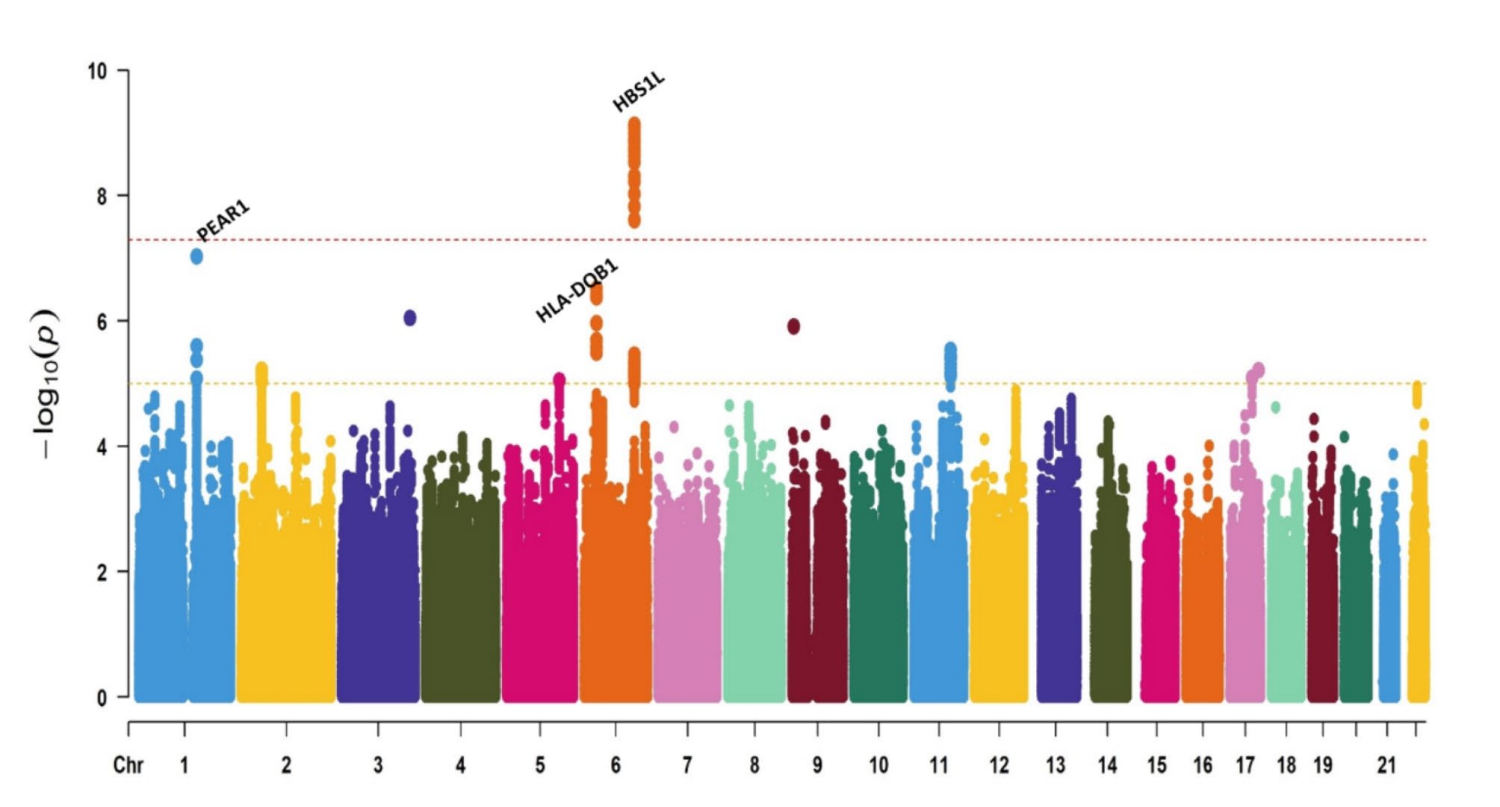
Manhattan plot of genome-wide significant association for FIB-4 index. A total of 1,476 cases of high-risk liver fibrosis (FIB-4 > 2.67) and 36,735 controls (FIB-4 < 1.3) of the general population of Taiwan were analyzed after quality control filtering. The genome-wide analysis was performed using logistic regression, assuming an additive model with age, age^2^, gender, tea and coffee consumption, vegetarian preference, alcohol consumption, physical activity, hepatitis serological markers, and the top ten principal components as covariates

**Fig. 3:**
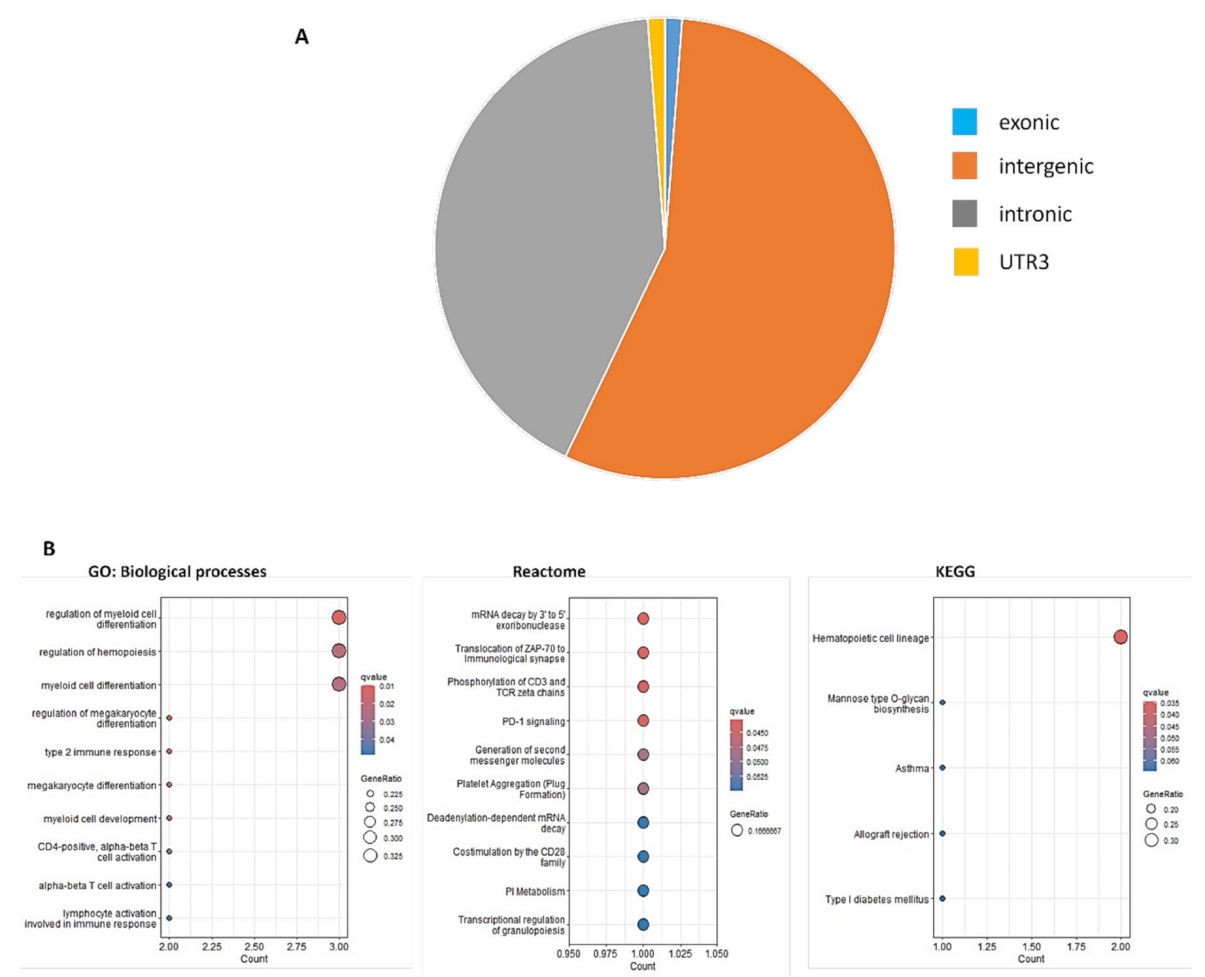
(**A**) Functional annotation of the GWAS suggestive loci of FIB-4 index in the general population of Taiwan. (**B**) The bubble plot of pathway enrichment analysis of GWAS suggestive variants. Data sources of gene ontology (GO): biological processes, Reactome, and KEGG. The bubble plot highlights the significant pathways that are enriched in the dataset. The red bubble indicates the most significant pathway enriched in the dataset, while the blue bubble suggests less significance

GWAS analysis detected 25 significant SNPs, which were all mapped to chromosome 6 (Fig. 2). The lists of the significant SNPs are shown in Supplementary Table 2. The Manhattan plot revealed a most significant locus on chromosome 6 (6q23.3), specifically at rs9399136, displaying a robust association with the reduced risk of FIB-4 index (OR = 0.72, *p* = 7.45 × 10^− 10^). According to the GWAS catalog (accessed on June 23, 2023), among these 25 variants, two of the identified variants, rs56293029 and rs9389269, had been previously associated with the FIB-4 index in alcoholic-related cirrhosis individuals with a protective effect. Additionally, 23 (rs9399136, rs9389268, rs9376091, rs9402685, rs9483788, rs7776054, rs4895440, rs9376092, rs35959442. rs9402686, rs9376090, rs4895441, rs34164109, rs9399137, rs35786788, rs9494145, rs7758845, rs9373124, rs9494142, rs6920211, rs11759553, rs6934903, rs6569992) variants were not previously linked to the FIB-4 index, signifying their novel role in preventing liver fibrosis. Most of the significant variants are located within the *HBS1-like translational GTPase* (*HBS1L*) and *HBS1L-MYB* (*MYB proto-oncogene*) locus. The results of the regional association analysis for the SNPs of interest are shown in Supplementary Fig. 3. Most suggestive variants were in the intergenic (56%) and intronic regions (42%). In contrast, a minor proportion of these variants was present in the exonic (1%) and UTR-3 (1%) regions, as illustrated in Fig. 3A. Expanding our investigation, we performed a conditional analysis through a stepwise model selection approach to identify independent signals from the suggestive variants within chromosome 6. Our findings reveal the presence of five independent variants, shown in Supplementary Table 3, with the most affected variant being rs9274407, which exhibited a substantial effect size of 1.27 (95% CI (0.05–1.15)), suggesting a significant role in the progression of liver fibrosis.

### SNP heritability and pathway analysis

We estimate the FIB-4 index’s SNP heritability to determine the extent to which the additive effect of SNP influences the phenotypic variation in liver fibrosis using the human default model in SumHer [18]. Our result indicates that the proportion of phenotypic variation in liver fibrosis identified by the FIB-4 index is 6% (SD = 0.007). We conducted pathway enrichment analysis on the genes associated with suggestive variants identified in this study. This analysis revealed significant enrichment in several key pathways, as shown in Fig. 3B. Enrichment patterns mainly related to immune-related responses and hematopoietic system function, such as regulation of myeloid cell differentiation and hemopoiesis, and myeloid cell differentiation, were revealed in the GO: BP. The Reactome database underscores pathways related to immune regulation (e.g., mRNA decay by 3’ to 5’ exoribonuclease, translocation of ZAP-70 to immunological synapse, phosphorylation of CD3 and TCR zeta chains). KEGG pathway analysis further emphasizes the importance of immune function pathways, significantly enriching hematopoietic cell lineage.

## Discussion

The World Health Organization (WHO) proposes using the FIB-4 index to identify liver fibrosis when serum panels or biopsies are unavailable [9]. The Taiwan Biobank study involved 1,476 cases defined as high risk and 36,735 controls defined as low risk for liver fibrosis based on the FIB-4 index. In Taiwan, 3.86% of the general population exhibited a FIB-4 index ≥ 2.67, with 33% over 60 years old. This indicates that older individuals are more susceptible to developing liver fibrosis. Sugiyama et al. conducted a study in the general Japanese population, showing that a FIB-4 index ≥ 2.67 was observed in 16.4% of individuals aged over 70 years [20], which mirrored the result observed in this Taiwanese cohort. Furthermore, a comprehensive analysis of large-scale data spanning three decades in the Japanese population revealed that, despite no significant alteration in the prevalence of a high FIB-4 index (≥ 2.67), individuals with a high FIB-4 index still demonstrated an elevated risk of developing liver cirrhosis and chronic hepatitis [21]. A previous study has indicated that the FIB-4 index exhibits age dependence in individuals with liver disease since the age parameter is included as the numerator in the FIB-4 index formula [22]. In cases where the exact disease onset is uncertain, age has previously been employed as a proxy to estimate disease duration, and it has shown a correlation with the presence of advanced fibrosis [23]. Diet preference might influence the progression of liver fibrosis [12].

Our multivariate analysis also showed that tea consumption is significantly associated with decreased risk of liver fibrosis. Therefore, we followed the analysis with GWAS to identify specific genetic susceptibility loci related to liver fibrosis by incorporating dietary patterns, including tea and coffee consumption and vegetarian preference, lifestyle (alcohol consumption, physical activity), and hepatitis serological markers (anti-HCV, HBsAg, and HBeAg) as covariates in this study. Our GWAS discovered 25 SNPs within the *HBS1L* and *HBS1L-MYB* genetic regions, including 23 novel SNPs had previously been reported in various studies, predominantly related to red blood cells (RBC), white blood cells (WBC), and platelet-related traits. The leading SNPs in this study were detected at 6p23.3 locus, including the top SNP rs9399136 in *HBS1L*, all showing protective effects of liver fibrosis. Although *HBS1L* was reported to be protective for liver fibrosis in previous GWAS [10], no clear evidence has been presented to comprehend the role of *HBS1L* in developing liver fibrosis. Several studies have proposed the function of intergenic variants of *HBS1L-MYB* in hematopoietic regulation, including fetal hemoglobin (HbF) and erythropoietin (EPO) levels [24, 25]. The expression of HbF ameliorated the inflammatory stress in a mouse model [26], while EPO reduces hepatic fibrosis by inhibiting the activation of hepatic stellate cells and macrophages and has a protective effect in various organs, including the liver, by interacting with pathways involved in inflammation, apoptosis, and fibrosis [27, 28]. This association implies a potential relationship between the *HBS1L* and its protective effect on liver fibrosis through anti-inflammation. In contrast, the most affected independent variant is rs9274407, within the *HLA-DQB1* gene, which has demonstrated a robust association with an increased risk of liver fibrosis (OR = 1.26, 95% CI 0.05–1.15), and it has been previously reported as a susceptibility variant with an increased risk of drug-induced liver injury [29]. Therefore, it can potentially be a novel variant associated with liver fibrosis. Interestingly, the observed effect of rs9274407 contradicted that of the lead SNP, rs9399136, in which rs9399136 showed a reduced risk of liver fibrosis. These two loci modify the risk of liver fibrosis in opposite directions. Human leukocyte antigen (HLA) regions have been identified as critical genetic markers for various liver illnesses through GWAS [30–32].

Dietary factors have shown their impact on the risk of liver fibrosis, especially tea intake, which was shown to have anti-inflammatory properties [12]. Several studies suggest dietary polyphenols can influence gene expression and metabolic pathways [13, 33]. A study in chronic hepatitis B patients revealed that higher coffee consumption was consistently linked to a reduced risk of elevated fibrosis markers [34]. The antioxidant-rich diet, particularly the consumption of tea and coffee, has been associated with beneficial effects on liver health. The anti-inflammatory properties might potentially influence the progression of liver fibrosis. This study included lifestyle information such as physical activity and alcohol consumption in the multivariate analysis, finding that alcohol intake increased the risk of liver fibrosis, which is consistent with the previous study [35]. In contrast, regular physical activity was linked to a reduced risk of liver fibrosis supporting the findings from UK Biobank cohort showed a 38% reduction in liver disease with 2,500 extra daily steps [36]. Viral infections, like hepatitis B and C, also elevate liver fibrosis risk by promoting collagen accumulation through activated hepatic stellate cells (HSCs) [37, 38].

In addition, this study revealed that the genes were significantly enriched for terms and pathways related to immune-related processes. Song, et al. showed that immune-related factors, such as interleukin-13 (IL-13), transforming growth factor beta (TGF-β), inflammatory markers, macrophages, and T-lymphocytes, contribute to liver fibrosis progression in mice [39]. In addition, previous studies indicate that immune cell-mediated activation of HSCs can promote hepatic fibrosis through the production of extracellular matrix proteins and suppression of natural killer (NK) cells’ anti-fibrotic functions [40, 41]. Some evidence﻿﻿ suggests that the HLA gene may play a role in liver fibrosis, but the exact mechanism is not well established. Studies showed that the HLA region is highly polymorphic and may confer a greater risk of MASH and MASH-related cirrhosis by regulating the immune response and the balance between inflammation and tissue repair in the liver [30, 42, 43].

The present study possesses several strengths, including using data from the general population of Taiwan biobank, which provides rich resources to identify genetic variations associated with the FIB-4 index. The FIB-4 index as a non-invasive marker was superior to seven other non-invasive fibrosis markers, such as the aspartate aminotransferase to platelet ratio index (APRI) and the NAFLD fibrosis score (NFS) [17]. Moreover, the FIB-4 index was demonstrated to be a more valuable predictor for the progression of chronic hepatitis and liver cirrhosis and minimize the need for liver biopsy in HIV/HCV-coinfected patients [21, 44]. This study is subject to certain limitations, notably the reliance on self-reported dietary information for tea, coffee, and vegetarian consumption. The absence of follow-up participant datasets in the present study restricts a comprehensive examination over time. While the FIB-4 index is a valuable biomarker for evaluating liver fibrosis severity, it may not accurately capture the tissue-specific processes involved in liver fibrosis development. Liver fibrosis is a complex process influenced by various genetics, environmental, and lifestyle factors. Relying solely on the FIB-4 index might overlook important genetic factors associated with the risk of fibrosis. Moreover, GWAS based on liver biopsy or additional liver image data are required to validate the functional impact of identified genetic variants and their role in fibrosis enhancement.

## Conclusions

Our findings revealed that more than one-third of individuals over 60 were at high risk of liver fibrosis. Furthermore, tea consumption shows a protective effect for liver fibrosis risk. In the GWAS analysis accounting for dietary factors, minor C allele at rs9399136 (*HBS1L*) was associated with a reduced risk of liver fibrosis, whereas the minor A allele at rs9274407 (*HLA-DQB1*) heightened the risk of liver fibrosis. Additionally, pathway analysis from GWAS variants suggested the potential significance of immune responses in the pathogenesis of liver disease.

## Electronic Supplementary Material

Below is the link to the electronic supplementary material.

Supplementary Material 1

## Data Availability

The datasets used and/or analyzed during the current study are available from the corresponding author upon reasonable request.

## Abbreviations

ARHGEF3: Rho guanine nucleotide exchange factor 3
COJO: Conditional and Joint
FIB-4: Fibrosis-4 Index
GWAS: Genome-wide association study
HBS1L: HBS1-like translational GTPase
HLA-DQB1: Major histocompatibility complex, class II, DQ beta 1
LD: Linkage disequilibrium
MRC1: Mannose receptor C-type 1
MYB: MYB proto-oncogene
NFS: NAFLD fibrosis score
SNP: Single nucleotide polymorphism
MASH: Metabolic dysfunction-associated steatohepatitis
TGF-β: Transforming growth factor beta
