# Supplementary material for: Dietary contributions in the genetic variation of liver fibrosis: a genome-wide association study of fibrosis-4 index in the liver fibrosis development
Source: Cell Biosci. 2024 Nov 22;14:141. doi: 10.1186/s13578-024-01321-6 (PMC11583755; doi:10.1186/s13578-024-01321-6)
Supplement: Supplementary file 1 — Supplementary Material 1 [file 13578_2024_1321_MOESM1_ESM.docx]

**Supplementary Table 1.** Clinical characteristics of participants included in the case-control genome-wide association study in Taiwan Biobank

| **Characteristic** | **Mean ± SD**  **N = 38,211** | **Control**  **N = 36,735** | **Case**  **N = 1,476** | **Reference value** |
| --- | --- | --- | --- | --- |
| Age | 46.65 ± 9.98 | 46.06 ± 9.62 | 61.48 ± 6.85 | - |
| Sex (male,%) | 30.18 | 29.77 | 40.45 | - |
| Platelet (1000/µL) | 263.80 ± 61.55 | 268.8 ± 56.76 | 138.8 ± 40.76 | 150 ~ 400 |
| AST (U/L) | 23.85 ± 13.21 | 22.93 ± 7.44 | 46.58 ± 51.03 | ≤ 34 |
| ALT (U/L) | 23.51 ± 20.40 | 22.86 ± 16.39 | 39.47 ± 61.86 | ≤ 36 |
| Triglyceride (mg/dL) | 116.1 ± 94.40 | 116.3 ± 93.61 | 111.0 ± 112.26 | < 150 |
| LDL-C (mg/dL) | 120.8 ± 31.88 | 121.2 ± 31.83 | 111.0 ± 31.66 | < 100 |
| HDL-C (mg/dL) | 54.23 ± 13.08 | 54.14 ± 12.95 | 56.27 ± 15.92 | Male (≥ 40), female (≥ 50) |
| Total cholesterol (mg/dL) | 194.8 ± 35.66 | 195 ± 35.57 | 189.8 ± 37.47 | < 200 |
| Tea consumption |  |  |  |  |
| No | 36,143 | 34,708 | 1,435 |  |
| Yes | 2,068 | 2,027 | 41 |  |
| Coffee |  |  |  |  |
| No | 35,727 | 34,308 | 1,419 |  |
| Yes | 2,484 | 2,427 | 57 |  |
| Vegetarian diet |  |  |  |  |
| No | 37,567 | 36,116 | 1,451 |  |
| Yes | 644 | 619 | 25 |  |
| Alcohol consumption |  |  |  |  |
| No | 36,267 | 34,922 | 1,345 |  |
| Yes | 1,944 | 1,813 | 131 |  |
| Physical activity |  |  |  |  |
| No | 12,625 | 11,734 | 891 |  |
| Yes | 25,586 | 25,001 | 585 |  |
| Anti-HCV |  |  |  | Negative |
| No | 37,516 | 36,181 | 1,335 |  |
| Yes | 695 | 554 | 141 |  |
| HBsAg |  |  |  | Negative |
| No | 34,575 | 33,399 | 1,176 |  |
| Yes | 3,636 | 3,336 | 300 |  |
| HBeAg |  |  |  | Negative |
| No | 37,955 | 36,500 | 1,455 |  |
| Yes | 256 | 235 | 21 |  |

Abbreviations: AST, aspartate transaminase; ALT, alanine transaminase; LDL-C, low-density lipoprotein cholesterol; HDL-C, high-density lipoprotein cholesterol. HBsAg, Hepatitis B surface antigen; HBeAg, Hepatitis B e antigen.

**Supplementary Table 2.** The significant SNPs (*p* < 5 × 10^− 8^) for the FIB-4 index

| CHR | SNP | BP | Ref | Alt | Nearest gene | Position | OR | *p-value* |
| --- | --- | --- | --- | --- | --- | --- | --- | --- |
| 6 | rs9399136 | 135081201 | T | C | *HBS1L* | Intergenic | 0.72 | 7.45 × 10^− 10^ |
| 6 | rs9389268 | 135098493 | A | G | *HBS1L* | Intergenic | 0.72 | 7.62 × 10^− 10^ |
| 6 | rs9376091 | 135098498 | C | T | *HBS1L* | Intergenic | 0.72 | 7.64 × 10^− 10^ |
| 6 | rs9402685 | 135098550 | T | C | *HBS1L* | Intergenic | 0.72 | 7.71 × 10^− 10^ |
| 6 | rs9483788 | 135114363 | T | C | *HBS1L-MYB* | Intergenic | 0.72 | 8.24 × 10^− 10^ |
| 6 | rs7776054 | 135097778 | A | G | *HBS1L* | Intergenic | 0.72 | 9.91 × 10^− 10^ |
| 6 | rs9389269 | 135106021 | T | C | *HBS1L-MYB* | Intergenic | 0.73 | 1.25 × 10^− 9^ |
| 6 | rs4895440 | 135105420 | A | T | *HBS1L-MYB* | Intergenic | 0.73 | 1.31 × 10^− 9^ |
| 6 | rs9376092 | 135106006 | C | A | *HBS1L-MYB* | Intergenic | 0.73 | 1.32 × 10^− 9^ |
| 6 | rs35959442 | 135103041 | C | G | *HBS1L* | Intergenic | 0.73 | 1.34 × 10^− 9^ |
| 6 | rs9402686 | 135106679 | G | A | *HBS1L-MYB* | Intergenic | 0.73 | 1.41 × 10^− 9^ |
| 6 | rs9376090 | 135090090 | T | C | *HBS1L* | Intergenic | 0.72 | 1.41 × 10^− 9^ |
| 6 | rs4895441 | 135105435 | A | G | *HBS1L-MYB* | Intergenic | 0.73 | 1.75 × 10^− 9^ |
| 6 | rs34164109 | 135100038 | C | T | *HBS1L* | Intergenic | 0.72 | 1.77 × 10^− 9^ |
| 6 | rs9399137 | 135097880 | T | C | *HBS1L* | Intergenic | 0.72 | 1.99 × 10^− 9^ |
| 6 | rs56293029 | 135097901 | C | A | *HBS1L* | Intergenic | 0.72 | 2.37 × 10^− 9^ |
| 6 | rs35786788 | 135097904 | G | A | *HBS1L* | Intergenic | 0.72 | 2.37 × 10^− 9^ |
| 6 | rs9494145 | 135111414 | T | C | *HBS1L-MYB* | Intergenic | 0.72 | 2.96 × 10^− 9^ |
| 6 | rs7758845 | 135107399 | A | C | *HBS1L-MYB* | Intergenic | 0.74 | 4.85 × 10^− 9^ |
| 6 | rs9373124 | 135102071 | T | C | *HBS1L* | Intergenic | 0.74 | 4.93 × 10^− 9^ |
| 6 | rs9494142 | 135110502 | T | C | *HBS1L-MYB* | Intergenic | 0.74 | 6.06 × 10^− 9^ |
| 6 | rs6920211 | 135110180 | T | C | *HBS1L-MYB* | Intergenic | 0.74 | 9.47 × 10^− 9^ |
| 6 | rs11759553 | 135101158 | A | T | *HBS1L* | Intergenic | 0.75 | 1.51 × 10^− 8^ |
| 6 | rs6934903 | 135130426 | T | A | *HBS1L-MYB* | Intergenic | 0.74 | 2.43 × 10^− 8^ |
| 6 | rs6569992 | 135131014 | G | A | *HBS1L-MYB* | Intergenic | 0.74 | 2.49 × 10^− 8^ |

Abbreviations: SNP, single nucleotide polymorphism; Chr, chromosome; Ref, Reference Allele; Alt, Alternative Allele; OR, odds ratio.

**Supplementary Table 3**. Conditional and Joint (COJO) analysis using GCTA on the suggestive variants

| SNP | Chr | Position | EA | OR (J) | SE (J) | *P*-value (J) | gene | LD-*r* |
| --- | --- | --- | --- | --- | --- | --- | --- | --- |
| rs9274407 | 6 | 32665055 | A | 1.26728 | 0.048994 | 1.61 × 10^− 147^ | *HLA-DQB1* | 0 |
| rs2210366 | 6 | 135094070 | A | 1.46656 | 0.054488 | 1.45 × 10^− 159^ | *HBS1L* | -0.46 |
| rs11759553 | 6 | 135101158 | T | 1.14232 | 0.071062 | 3.83 × 10^− 58^ | *HBS1L* | 0.64 |
| rs6569992 | 6 | 135131014 | A | 0.99902 | 0.072233 | 1.67 × 10^− 43^ | *MYB, HBS1L* | -0.34 |
| rs10457633 | 6 | 135208073 | A | 0.98248 | 0.052909 | 5.69 × 10^− 77^ | *-* | 0 |

Abbreviations: SNP, single nucleotide polymorphism; Chr, chromosome; EA, Effect Allele; OR(J), odds ratio joint analysis; SE(J), standar error joint analysis, *P*-value(J), *P*-value joint analysis; LD-*r*, linkage disequilibrium correlation between variants.

**
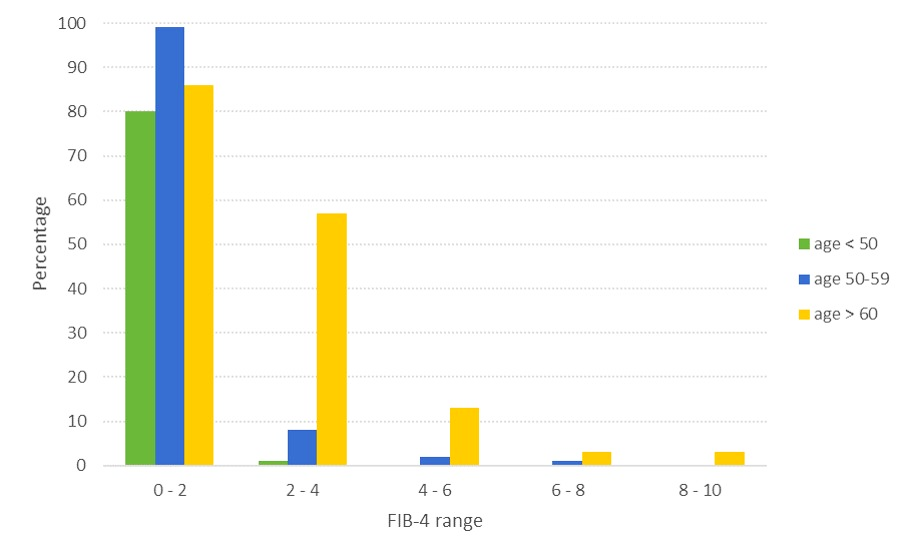
**

**Supplementary Fig. 1.** The proportion of the FIB-4 index in each age group in the general population of Taiwan (N = 38,211). Age > 60 years had a high FIB-4 index value.


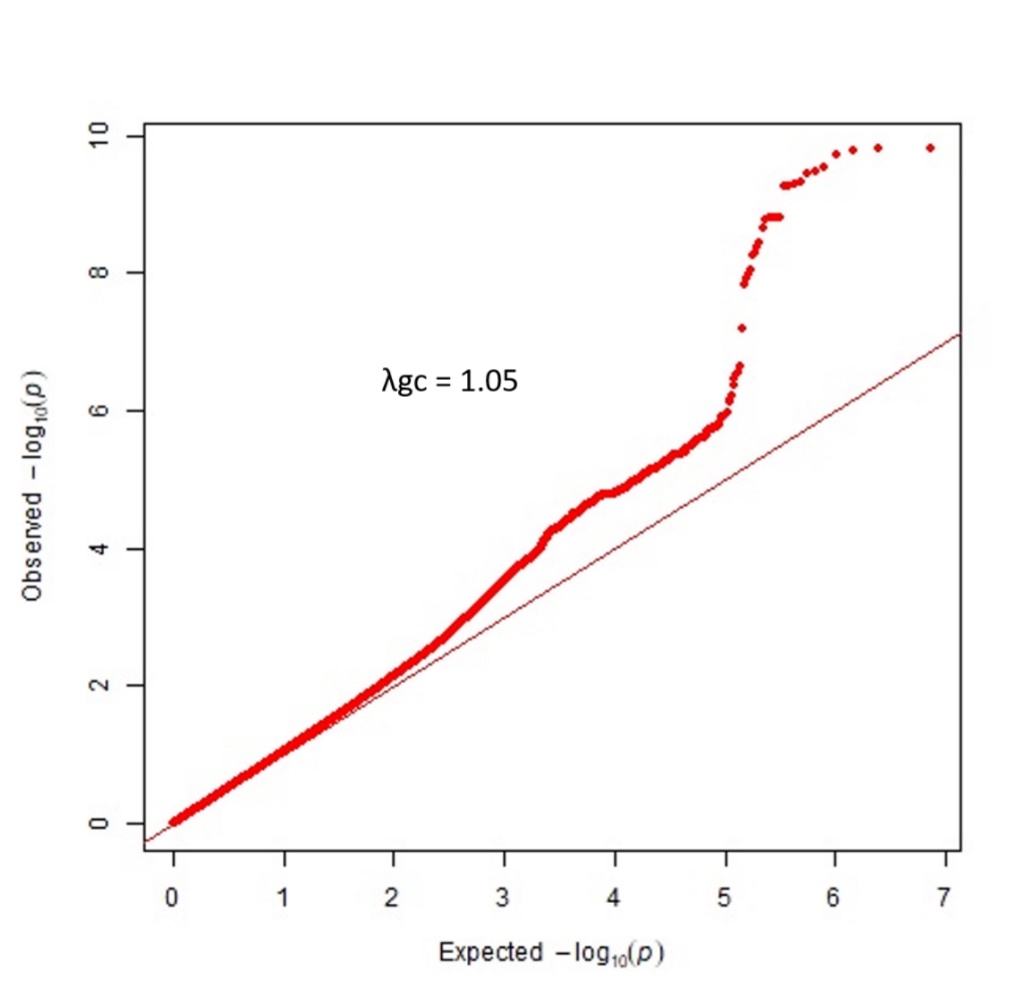


**Supplementary Fig. 2.** Q-Q plot of genome-wide significant association for FIB-4 index. Genomic inflation factor = 1.05 indicated no population stratification.


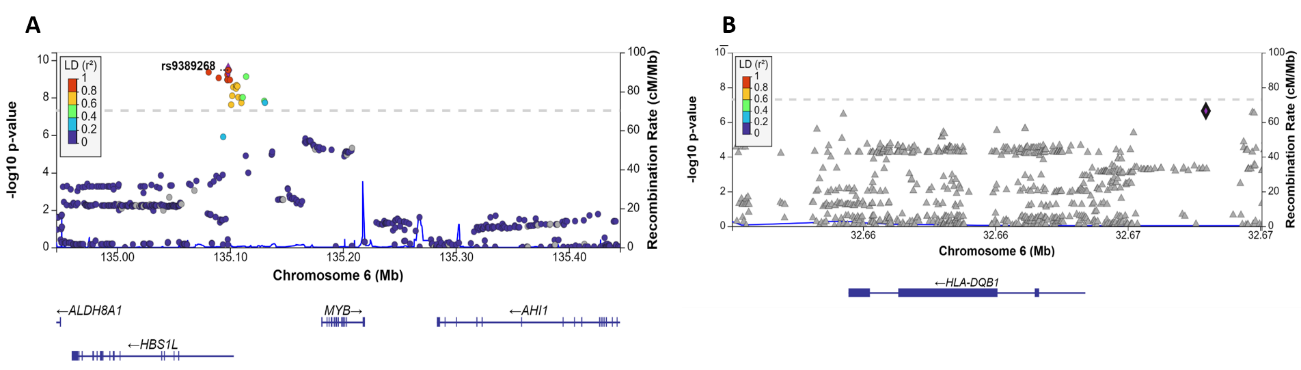


**Supplementary Fig. 3**. Regional plots were created using LocusZoom for the GWAS on the FIB-4 index in the general population of Taiwan. (A) Regional plot of the GWAS significant variants. (B) Regional plot of the independent variant identified by conditional analysis. The color represented the extent of linkage disequilibrium (LD) structure. LD was calculated based on Genome Build GRCh38 East Asian (EAS) Ancestry. Variants missing LD information were colored in grey. The lead SNPs within each region were marked with a diamond symbol.
